# Functional assays to screen and select monoclonal antibodies that target *Yersinia pestis*

**DOI:** 10.1080/21645515.2023.2216085

**Published:** 2023-06-08

**Authors:** Sergei S. Biryukov, Nathaniel O. Rill, Christopher P. Klimko, Jennifer L. Dankmeyer, Jennifer L. Shoe, Melissa Hunter, Yuli Talyansky, Derrick Hau, Marcellene A. Gates-Hollingsworth, Sujata G. Pandit, David P. Fetterer, Ju Qiu, Michael L. Davies, David P. AuCoin, Christopher K. Cote

**Affiliations:** a Bacteriology Division, United States Army Medical Research Institute of Infectious Diseases (USAMRIID), Frederick, MD, USA; b Department of Microbiology and Immunology, University of Nevada, Reno School of Medicine, Reno, NV, USA; c Biostatistics Division, United States Army Medical Research Institute of Infectious Diseases (USAMRIID), Frederick, MD, USA

**Keywords:** *Yersinia pestis*, mice, immunity, antibody treatment, antibody screening, plague

## Abstract

*Yersinia pestis* is a gram-negative bacterium that causes plague in animals and humans. Depending on the route of disease transmission, the bacterium can cause an acute, often fatal disease that has a narrow window for treatment with antibiotics. Additionally, antibiotic resistant strains have been identified, emphasizing the need for novel treatments. Antibody therapy is an appealing option that can direct the immune system to clear bacterial infections. Advances in biotechnology have made both engineering and producing antibodies easier and more affordable. In this study, two screening assays were optimized to evaluate the ability of antibodies to promote phagocytosis of *Y.* *pestis* by macrophages and to induce a cytokine signature in vitro that may be predictive of protection in vivo. We evaluated a panel of 21 mouse monoclonal antibodies targeting either the anti-phagocytic capsule F1 protein or the LcrV antigen, which is part of the type 3 secretion system that facilitates translocation of virulence factors into the host cell, using two functional assays. Anti-F1 and anti-LcrV monoclonal antibodies both increased bacterial uptake by macrophages, with greater uptake observed in the presence of antibodies that were protective in the mouse pneumonic plague model. In addition, the protective anti-F1 and anti-LcrV antibodies produced unique cytokine signatures that were also associated with in vivo protection. These antibody-dependent characteristics from in vitro functional assays will be useful in down-selecting efficacious novel antibodies that can be used for treatment of plague.

## Introduction

*Yersinia pestis* is a gram-negative bacteria that is classified as a Tier 1 select agent by the United States Department of Health and Human Services due to its ability to cause rapidly fatal infections in both humans and animals. While bubonic plague is the most common form of the disease, pneumonic plague is a greater concern in the context of biodefense strategies.^1,2^ *Y. pestis* is a major biothreat due to its capacity for aerosol dissemination and its contagious nature in the pneumonic form. The illness can be treated with several different classes of antibiotics, including aminoglycosides (e.g., streptomycin) and fluoroquinolones (e.g., ciprofloxacin), if accurate diagnosis is made early after infection and antibiotic therapy is initiated without delay.^3^ However, antibiotic treatment options could become limited if the bacteria are engineered to become resistant or naturally acquire antibiotic resistance. The recent plague outbreak in Madagascar, which included cases of naturally acquired antibiotic resistance, emphasizes the need for novel therapeutics that can be used either alone or in combination with other medical countermeasures.^4–7^

Two protective antigens have been used to make subunit vaccines, including the Fraction 1 (F1) and the low-calcium response V (LcrV) antigens.^8,9^ The F1 protein is encoded by the *caf1* gene located on a large pMT plasmid and is expressed at 37°C. It inhibits the uptake of the bacteria by macrophages, creating an anti-phagocytic capsule.^10^ It is also thought to play a role in bacterial transmission because it was shown to inhibit the adhesion of the bacteria to human epithelial cells.^11^ However, strains of *Y. pestis* (e.g., C12 strain) have been identified that are F1-negative and still retain virulence in mice,^12–15^ emphasizing the need to additionally target another conserved and protective antigen that would be found in F1-negative strains, such as the LcrV protein.^16^ LcrV is encoded on the pCD1 plasmid and is a major virulence factor that is necessary for proper assembly of the translocation pore of the type 3 secretion system (T3SS) injectisome complex. This antigen facilitates translocation of the virulence factors called Yops into target cells, which results in the inhibition of phagocytosis and induction of apoptosis.^17^ The LcrV antigen has also been demonstrated to be a multifunctional protein as it has been shown to have immunomodulatory effects in both in vivo and in vitro settings as well as the ability to translocate into host cells.^18–20^ The protective epitope of the LcrV antigen has been mapped by several groups and includes amino acids 135 to 275.^17,21^

Active immunization with recombinant LcrV protein has been shown to confer protection against both bubonic and pneumonic models of plague caused by either an encapsulated (F1+) strain CO92 or non-encapsulated (F1-) strain C12,^22^ although the level of protection against non-encapsulated strains remains equivocal. Combining both F1 and LcrV antigens resulted in improved protection in mice infected with *Y. pestis*.^16^ Targeting these two antigens increases the likelihood of protecting against emerging or engineered *Y. pestis* isolates that may be F1-negative despite the known heterogeneity among the LcrV proteins from different isolates.^23,24^ Researchers in the United States have predominantly pursued a recombinant fusion-protein strategy (i.e., F1-V),^25–27^ whereas researchers in the United Kingdom focused their efforts on a vaccine with both distinct protein entities (i.e., F1 + V).^28–31^ However, to date, there is no FDA-approved vaccine against plague.

Vaccine studies using the F1 and LcrV antigens suggest that antibodies play a role in the acquired protection. In addition, passive protection has been accomplished using antibodies directed against either antigen.^21,30,32–38^ Passive immunization with two monoclonal antibodies (mAb) generated against *Y. pestis* LcrV (mAb 7.3) or F1 (mAb F1–04-A-G1) antigens are highly protective in mouse models of bubonic and pneumonic plague when administered prophylactically or 48 hours post-infection, either alone or in combination. Furthermore, when provided in combination, they exhibited a synergistic effect.^33,39^ The in vivo protection with the anti-LcrV antibody has been shown to correlate in vitro with increased phagocytosis by macrophages and reduced macrophage cell death following infection with *Y. pestis*.^17^

To screen and down-select antibodies generated against *Y. pestis*, two functional assays were optimized. These include the following: 1) gentamicin protection assay to quantitate intracellular and macrophage-associated *Y. pestis* using single plate-serial dilution spotting;^40^ and 2) multiplex bead-based immunoassay to quantitate cytokine/chemokine levels in the supernatants of *Y. pestis* infected macrophages. The phagocytosis and cytokine/chemokine assays are moderate to high-throughput and the assays can be performed in parallel and thus, can be used to screen antibodies and down-select or prioritize antibodies based on their activity in these functional assays. A total of 21 murine monoclonal antibodies (mAb) were generated against either F1 or LcrV antigen and were selected for testing in the optimized assays. F1–04-A-G1 is an IgG1 mAb that was generated to F1 capsule material and has been demonstrated to protect mice against plague.^33,41^ The anti-LcrV mAb 7.3 is also an IgG1 subclass antibody and is reliably protective in mouse models of plague.^21,39^ Due to their excellent passive protection in mice against *Y. pestis* challenge, anti-F1 F1–04-A-G1 and anti-LcrV 7.3 were used as benchmarks in testing the 21 novel mAbs with unknown protection profiles. Both F1–04-A-G1 mAb and 7.3 mAb increased phagocytosis of *Y. pestis* by macrophages and produced unique cytokine/chemokine signatures that were associated with in vivo protection of mice. These data were used to interpret results from our assays and guided our selection of criteria to predict which mAbs would be protective in mice exposed to aerosolized *Y. pestis*.

## Materials and methods

### Bacterial strains and growth conditions

The *Y. pestis* CO92 strain used for in vivo studies was isolated in sputum from a human case of pneumonic plague.^42^ To prepare challenge material, *Y. pestis* CO92 was plated on tryptose blood agar base slants and incubated for approximately 48 h at 28–30°C. The slants were then washed with heart infusion broth (HIB) medium supplemented with 0.2% xylose (HIBX), and the suspensions were adjusted to an OD_620_ of 1. The *Y. pestis* inoculum collected from the slants was then diluted 1:100 into HIBX and incubated for approximately 24 h at 28–30°C shaking at 150 rpm. The next day the bacteria were harvested by centrifugation and resuspended in HIB prior to the aerosolization protocol.

The *Y. pestis pgm*- pPst- strain, used in the in vitro assays, was generated at the United States Army Medical Research Institute of Infectious Diseases (Frederick, Maryland) and was kindly provided by Susan Welkos. *Y. pestis pgm*- pPst- is an attenuated strain derived from CO92, which is cured of the pPst plasmid containing the plasminogen activator virulence locus (*pla*) and is pigmentation-deficient due to the deletion of the 102 kb pigmentation locus (*pgm*).^43^ *Y. pestis pgm*- pPst- was grown on Remel® Sheep Blood Agar (SBA) plates (Thermo Scientific, Pittsburgh, PA) and incubated at 28°C or 37°C for 24 h. Bacterial colonies were harvested and used to inoculate 10 mL of brain heart infusion (BHI) broth (BD Biosciences San Jose, CA) which was then incubated for 2 h at 37ºC with shaking at 200 rpm prior to infecting the macrophages. To ensure the bacteria were harvested in the log phase of growth, the OD_600_ of the culture post-incubation was not allowed to exceed 1prior to incubation with antibodies.

### Mouse monoclonal antibodies

The anti-F1 mouse mAb F1–04-A-G1 was provided by James Burans and Jennifer Aldrich (Naval Medical Research Center, Silver Spring, MD).^33^ The anti-LcrV mouse mAb 7.3 was provided by Jim Hill (formerly of DSTL, Porton Down, Wiltshire, UK).^21,44^ The panel of 21 anti-F1 and anti-LcrV mAbs were recently developed and characterized by the AuCoin laboratory (University of Nevada, Reno, NV).^45^ Purified polyclonal mouse IgG (Rockland Immunochemicals, Limerick, PA) served as nonspecific control.

### Cell culture

RAW264.7 murine macrophage-like cells derived from an Abelson murine leukemia virus tumor (ATCC TIB-71) were grown at 37°C in 5% CO_2_ in low glucose DMEM containing 10% fetal bovine serum, 1% L-glutamine, 1% non-essential amino acids, and 1% HEPES buffer. Cells were used before passage 15 and seeded in 96-well plates.

### *Quantification of viable intracellular* Y. pestis *(Gentamicin protection assay)*

**Figure 1.**
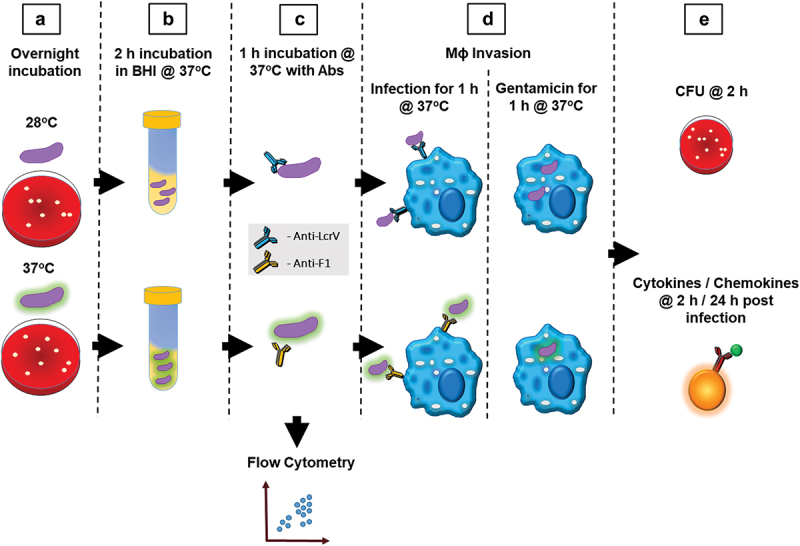
Gentamicin protection assay. (a) Initial growth of *Y. pestis pgm*- pPst was on sheep blood agar at 28°C (non-encapsulated) or 37°C (robust capsule) for 24 h. (b) Bacterial colonies were harvested and used to inoculate 10 mL of brain heart infusion (BHI) broth and incubated in BHI for 2 h at 37ºC with shaking at 200 rpm. (c) Bacterial concentration was adjusted to 8 x 10^6^ CFU/mL and incubated with 10 µl/mL of mAbs for 1 h at 37ºC. Flow cytometry was utilized to evaluate changes in bacteria cell morphology and aggregation. (d) RAW264.7 cells were infected, at an MOI of 10 and centrifuged at 200 x g for 5 minutes to initiate infection and incubated for 1 h at 37ºC. Gentamicin (8 µg/mL) was added to all wells to kill extracellular bacteria, and plates were incubated for an additional 1 h at 37°C. (e) At 2 h postinvasion intracellular *Y. pestis* was quantified using single plate-serial dilution spotting. Cytokine and chemokine panel was run on supernatant from *Y. pestis* infected macrophages 2 h or 24 h postinfection.

Bacterial cultures grown in BHI were suspended in DMEM, and multiplicity of infection (MOI) was estimated using an OD_600_ of 1. For macrophage infection assays, RAW264.7 cells were seeded into 96-well plates (1.5 × 10^4^ cell/well) one day prior to infection. *Y. pestis pgm*- pPst- at 8 × 10^6^ CFU/mL was pre-incubated with 10 µg/mL antibodies in DMEM for 1 h at 37°C prior to infection. Macrophages were then infected at an MOI of 10 in triplicate wells. The plates were centrifuged at 200 × *g* for 5 min to initiate infection and then placed into a 37°C incubator with 5% CO_2_. After 1 h of incubation, gentamicin (8 µg/mL) was added to the wells to kill the majority (>99%) of the extracellular bacteria, and the plates were incubated for an additional hour at 37°C incubator with 5% CO_2_.^46^ After incubation, the supernatant was aspirated and retained for further cytokine evaluation (Figure 1), while the macrophages were washed two times in PBS and lysed using 0.1% Triton X-100 in PBS. Serial dilutions of lysates were plated, by spotting 5 µL in duplicate, on sheep’s blood agar (SBA) and incubated for 16–18 h at 28°C for CFU enumeration (Figure 1). The geometric means of CFU per mL were plotted for control samples not incubated with antibody compared to those incubated with 10 µg/mL of each antibody. For evaluation of cytokines 24 h post-infection, medium with gentamicin at 8 µg/mL was replaced with medium with gentamicin at 2 µg/mL at 2 h post-invasion (Figure 1).^46^

### Flow cytometry

*Y. pestis pgm*- pPst- log cultures, at approximately 8 × 10^6^ CFU/mL, were incubated with 10 µg/mL of antibody in DMEM as described above. The samples were centrifuged at 10,000 × g for 10 min and resuspended in 300 µL 0.85% NaCl. Samples were washed and labeled at room temperature with LIVE/DEAD BacLight staining reagent (Thermo Fisher) to identify live *Y. pestis* cells as positive for SYTO 9 dye. Samples were read on a FACSCanto II (BD Biosciences, San Jose, CA) flow cytometer and analyzed using FlowJo v10.8.1 (BD Biosciences, Ashland, OR) software to determine each antibody’s effect on bacterial aggregation. *Y. pestis* aggregates were identified in the forward versus side scatter (FSC vs SSC) dot plot, followed by gating on live cells in the (SYTO 9 vs propidium iodide) dot plot, and then using the (FSC vs SSC) dot plot to categorize bacterial aggregates as small, medium or large.

### Quantification of cytokines by Luminex

The supernatants from *Y. pestis pgm*- pPst- infected and uninfected RAW264.7 cells in the presence or absence of antibodies were evaluated for cytokine expression by Luminex MagPix 36-plex mouse panel as per manufacturer’s directions (Thermo Fisher Scientific, Grand Island, NY) and as shown in Figure 1. The levels (pg/mL) of the following 36 cytokines/chemokines were measured: Eotaxin, ENA-78/CXCL5, G-CSF, GM-CSF, GRO-α,IFN-α, IFN-γ, IL-1α, IL-1β, IL-2, IL-4, IL-5, IL-6, IL-9, IL-10, IL-12 (p70), IL-13, IL-15/IL-15 R, IL-17A, IL-18, IL-22, IL-23, IL-27, IL-28, IL-31, IP-10, LIF, M-CSF, MCP-1, MCP-3, MIG, MIP-1α, MIP-1β, MIP-2, RANTES, and TNF-α. Only cytokines that exhibited elevated or depressed levels relative to *Y. pestis*-only infected macrophages were shown. Heat maps were prepared which showed the fold-change as an expression of each cytokine/chemokine relative to the *Y. pestis* only infection, as detailed in the table footnotes.

### Exposure of mice to *Y.* *pestis* challenge

Female, 6- to 8-week-old BALB/c mice were purchased from Charles River Laboratories (Frederick, MD). Mice were administered intraperitoneal injections of each antibody in 200 µL of PBS, 18 h prior to challenge. For the pneumonic plague mouse model, aerosolization challenge doses of *Y. pestis* strain CO92 were prepared as previously described.^27,47–49^ Mice were transferred to wire mesh cages and placed in a whole-body aerosol chamber within a Class 3 biological safety cabinet located inside a BSL-3 laboratory. There they were exposed to aerosols (~1–3 µm in diameter) of *Y. pestis* created by a three-jet Collison nebulizer. To determine the inhaled bacterial dose, samples were collected from an all-glass impinger (AGI) vessel and analyzed by CFU calculations.

### Statistics

Gentamicin protection assay results (CFU and cytokine data) were compared by an exact two-sided Wilcoxon test on values that were log-transformed for analysis and were summarized as median and IQR (Q1, Q3), geometric means and geometric standard error. The cytokines were stratified by date of experiments. The log-rank test was used to compare mouse survival curves post-challenge and time-to-death (TTD). The mortality rates were evaluated with the Fisher's exact test. The analysis was implemented in SAS version 9.4 (SAS Institute Inc., Cary, NC).

## Results

### Y. pestis *infection assay optimization*

To screen and identity antibodies that help eliminate *Y. pestis* through inducing phagocytosis (and possibly bactericidal activity) and/or cytokine secretion, we exploited different bacterial growth conditions in our in vitro assays. The temperature and time of bacterial growth affects the expression of various *Y. pestis* proteins. In particular, the F1 and the LcrV proteins are both induced at mammalian body temperature. Depending upon the targeted protein, it is necessary to use *Y. pestis* grown at specific temperatures to adequately characterize antibody-bacteria interactions in vitro. *Y. pestis* requires at least 4 h of growth at 37°C prior to infection to produce enough capsule to prevent phagocytosis,^10^ while only 30 min at 37°C is sufficient to induce T3SS-associated proteins known to play a role in the inhibition of phagocytosis.^50–52^

To optimize the availability of selective temperature-dependent virulence factors, *Y. pestis pgm*- pPst- was cultured under different temperature conditions, followed by incubation in the presence of antibody prior to performing a gentamicin protection assay (Figure 1) to discern whether the antibody enhanced bacterial phagocytosis by macrophages. *Y. pestis* was grown at 28°C or 37°C on SBA plates for 24 h, followed by a switch to liquid BHI medium for an additional 2 h of growth at 37°C with shaking at 200 rpm. *Y. pestis pgm-* pPst- was then incubated with 10 µg/ml of antibodies for 1 h prior to infection of macrophages. Macrophages that were infected with *Y. pestis* grown at 28°C on SBA and treated with 10 µg/mL of anti-LcrV mAb 7.3 displayed higher levels of bacterial internalization and intracellular bacterial load after 2 h, due to the induction of LcrV during the brief 37°C incubation and the lack of a robust capsule that may potentially limit access to the T3SS injectisome (Figure S1a). No enhancement in bacterial uptake was observed in the presence of F1–04-A-G1 mAb or control mIgG under these growth conditions (Figure S1a). Conversely to this, extensive bacterial internalization and intracellular bacterial load were present after 2 h in macrophages infected with *Y. pestis* grown at 37°C on SBA and treated with 10 µg/mL of anti-F1 mAb F1–04-A-G1 (Figure S1b). Of note, the level of bacterial invasion is drastically diminished with *Y. pestis* grown at 37°C relative to *Y. pestis* grown at 28°C on SBA partly due to the F1 capsule formation reducing both the adhesion to and phagocytosis by macrophages (Figure S1a vs b).^53^ In subsequent experiments, anti-F1 mAbs were characterized employing the *Y. pestis* grown at 37°C, while *Y. pestis* initially grown at 28°C, to limit F1 capsule formation and increase LcrV production and availability, was utilized for anti-LcrV mAbs.

### Anti-F1 mAbs, but not anti-LcrV mAbs, increase bacterial aggregation in vitro

Utilizing these optimized *Y. pestis* growth conditions, we tested novel anti-F1 and anti-LcrV monoclonal antibodies to determine their effect on bacterial aggregation and phagocytosis by macrophages. Twenty-one novel anti-*Y. pestis* antibodies, with unknown in vivo protection profiles, were compared to the well-characterized protective anti-F1 mAb F1–04-A-G1 mAb and anti-LcrV mAb 7.3. As above, monoclonal antibodies directed against F1 or LcrV antigens were incubated at 10 µg/mL with *Y. pestis pgm*- pPst- for 1 h.

**Figure 2.**
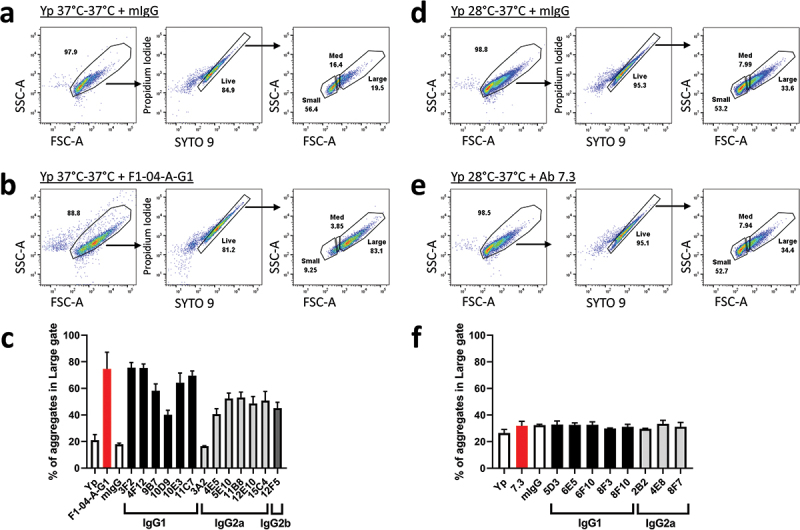
Characterizing aggregation associated with antibody interactions with *Y. pestis. Y. pestis pgm*- pPst- cells (approximately 8 x 10^6^ cells/mL) were treated with 10 µg/mL of the specified antibody and labelled with LIVE/DEAD BacLight staining reagent. Depicted is the gating strategy used to designate live cell aggregates as small, medium or large by forward scatter (FSC-A) and side scatter (SSC-A). (a-b) Examples of gating strategy for *Y. pestis* grown overnight at 37°C followed by 2 h at 37°C in BHI, incubated with negative control mIgG (A) or established anti-F1 clone F1–04-A-G1 (b). (c) The percent of live aggregates categorized as “Large” was determined for each anti-F1 mAb, compared to F1–04-A-G1 and negative control mIgG. (d-e) Examples of gating strategy for *Y. pestis* grown at 28°C overnight followed by 2 h at 37°C in BHI, incubated with negative control mIgG (d) or established anti-LcrV clone 7.3 (e). (f) The percent of live aggregates categorized as “Large” was determined for each anti-LcrV mAb, compared to 7.3 and negative control mIgG. Bars represent the mean and error bars represent the SEM of three independent experiments.

To determine each antibody’s effect on bacterial aggregation, we performed flow cytometry to observe the size of particles in solution after the bacteria had been incubated for 1 h with antibody. Figures 2a,b show the gating strategy used to identify bacterial particles and gate on live cells. All anti-F1 antibodies increased the size of bacterial aggregates, with the exception of 3A2 mAb which was similar to control mIgG (Figure 2c). Four of the novel antibodies (3F2, 4F12, 10E3, and 11C7) had comparable aggregating properties to anti-F1 mAb F1–04-A-G1 mAb. No antibodies targeting LcrV (including the benchmark anti-LcrV mAb 7.3) showed significant increase in aggregation above control mIgG or *Y. pestis* alone (Figure 2d).

### Both anti-F1 and anti-LcrV mAbs increase bacterial uptake by RAW264.7 cells in vitro

**Figure 3.**
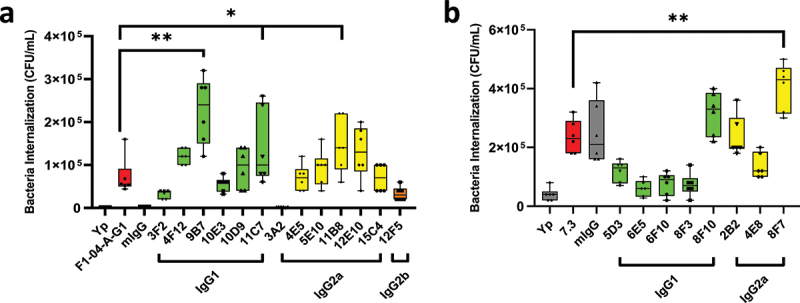
Pretreatment of *Y. pestis* with anti-F1 or anti-LcrV mAbs produces marked invasion enhancement of macrophages. *Y. pestis pgm*- pPst- initially grown at a) 37°C or b) 28°C on SBA for 24 h followed by a switch to liquid BHI broth and grown for an additional 2 h at 37°C. RAW264.7 cells were infected, at an MOI of 10, after 1 h pretreatment of *Y. pestis* with (a) anti-F1 mAbs or (b) anti-LcrV mAbs at 10 μg/ml. Macrophages infected with *Y. pestis* (Yp) alone or with mouse non-specific polyclonal IgG (mIgG) were used as controls. The number of viable bacteria recovered after 2 h of infection from one representative experiment (*n* = 6) are shown. The box-plots depict the median value, each technical replicate in that iteration and the 1^st^ and 3^rd^ quartile values. This is a representative experiment of at least four similar experiments, *n* = 6 per experiment. Statistical significance was determined using two-sided, two-sample Wilcoxon test comparisons and a *p*-values of *p ≤ .05, **p ≤ .01 were denoted as significant relative to F1–04-A-G1 or 7.3 mAbs.

To determine each antibody’s effect on phagocytosis by macrophages, we performed the gentamicin protection assay as described above. All anti-F1 antibodies enhanced the level of bacterial internalization into macrophages (*p* < .005 vs *Y. pestis* alone), with the exception of 3A2 mAb which was similar to the control mIgG (*p* = .08 vs mIgG) (Figure 3a). Of note, three novel anti-F1 mAbs (9B7, 11C7 and 11B8) significantly enhanced the level of invasion relative to F1–04-A-G1 mAb (*p* < .05 vs F1–04-A-G1) (Figure 3a).

All anti-LcrV antibodies enhanced the level of bacterial internalization into macrophages, p < .01 vs *Y. pestis*, with the exception of 6E5, 6F10, and 8F3 mAbs, which were not found to be statistically significant relative to *Y. pestis* alone (Figure 3b). Interestingly, the commercially available normal mouse IgG used as a negative control significantly enhanced the uptake of *Y. pestis* cells initially grown at 28°C, suggesting cross-reactivity with antigens on the surface of the bacteria that are normally obscured by capsular material. Of the eight novel anti-LcrV mAbs, 8F7 significantly enhanced the level of invasion relative to the benchmark 7.3 mAb (*p* < .01) (Figure 3b). There was no significant difference on the level of bacterial invasion between antibody subclasses (IgG1, IgG2a, and IgG2b) for either the anti-LcrV or anti-F1 mAbs. Of note, both anti-F1 and anti-LcrV mAbs were able to opsonize bacteria for phagocytosis, although only anti-F1 mAbs led to aggregation of the bacteria, likely because *Y. pestis* is coated with F1 capsule antigen while LcrV on the bacterial cell-surface it is limited to relatively sparsely distributed injectisomes.^19,20,54–56^

### Different monoclonal antibodies result in different cytokine signatures infected RAW264.7 cells in vitro

To further characterize these novel antibodies and look for correlations of in vivo protection, we sought to establish a predictive signature of cytokine and chemokine expression after invasion of macrophages. The unique cytokine and chemokine signatures of the novel mAbs were compared to the benchmark 7.3 and F1–04-A-G1 mAbs that are known to be highly protective against both bubonic and pneumonic plague. The putative protective signatures of the benchmark mAbs were identified as a combination of at least a two-fold or greater change in cytokine or chemokine expression relative to *Y. pestis*-only infected macrophages and a *p*-value of < .01.

Data collected 2 h after infection indicated that macrophages infected with *Y. pestis* pre-incubated with anti-LcrV 7.3 mAb (IgG1) significantly upregulated G-CSF, LIF, MIP-1β, TNF-α, MIP-1α, MCP-3, and MIP-2, while IL-2 and IL-28 were downregulated (Table 1). Twenty-four hours post-invasion, there was a significant upregulation of IL-10, LIF, M-CSF, IL-6, IL-17A, and IL-27, while Gro-α was downregulated (Table 2). Of the eight anti-LcrV antibodies, only 8F10 and 8F7 mAbs induced similar cytokine and chemokine profiles compared to 7.3 mAb. Four of the five IgG1 antibodies (5D3, 6E5, 6F10, and 8F3) had a similar cytokine response compared to macrophages infected with *Y. pestis* only. Two of the three IgG2a antibodies, 2B2 and 4E8, appeared to have a similar profile as the nonspecific control (mIgG). Based on the cytokine response (Table 2) and CFU data (Figure 3b) we predicted that 8F10 and 8F7 mAbs would be the most effective anti-LcrV candidate antibodies at providing protection in vivo.

**Table 1.** Fold change in cytokine levels 2 h post-invasion with *Y. pestis pgm*- pPst- initially grown at 28°C preincubated with anti-LcrV mAbs.

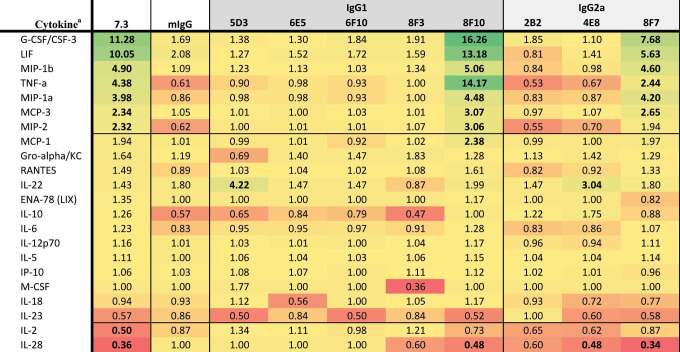

^a^The cytokine results are shown as the ratio of the levels expressed by Yp infected macrophages only and are based on the geometric mean (pg/mL). Cytokines where all values were identical, or all but one were identical, were omitted. Bolded values are up- or down-regulated compared to Yp only (up- or down-regulated >2-fold with *p*-value < .01).
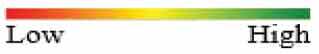

**Table 2.** Fold change in cytokine levels 24 h post invasion with *Y. pestis pgm*- pPst- initially grown at 28°C preincubated with anti-LcrV mAbs.

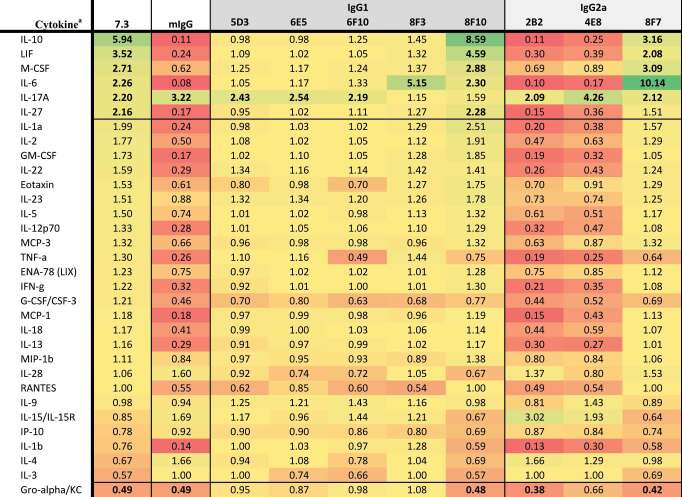

^a^The cytokine results are shown as the ratio of the levels expressed by Yp infected macrophages only and are based on the geometric mean (pg/mL). Cytokines where all values were identical, or all but one were identical, were omitted. Bolded values are up- or down-regulated compared to Yp only (up- or down-regulated >2-fold with *p*-value < .01).
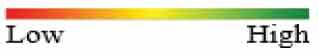

Cytokine and chemokine responses were also evaluated in *Y. pestis*-infected macrophages pre-incubated with F1–04-A-G1 mAb (IgG1) or the anti-F1 candidate antibodies. At 2 h after macrophage infection, G-CSF, MIP-2, and TNF-α were significantly downregulated (along with LIF, but significance was not reached for LIF due to variance within groups) (Table 3). All 13 anti-F1 mAbs produced a similar cytokine response compared to F1–04-A-G1, with the exception of 3A2 (IgG2a) which had elevated MIP-2 and TNF-α. Aside from 3A2, all IgG2a mAbs (4E5, 5E10, 11B8, 12E10, and 15C4) shared the F1–04-A-G1 pattern of downregulating G-CSF, MIP-2, and TNF-α, although interestingly all five led to significantly less MIP-2 and TNF-α and more G-CSF than F1–04-A-G1 (*p* < .05) (Table 3). At 24 h after macrophage infection, IL-10, LIF, GM-CSF, IL-6, IL-27, IL-2, TNF-α, and IL-12p70 were significantly downregulated (Table 4). Again, a similar signature was seen for all of the experimental anti-F1 antibodies with the exception of 3A2 (IgG2a). Furthermore, much like the pattern at the 2-h time point, the remaining IgG2a mAbs (4E5, 5E10, 11B8, 12E10 and 15C4) exhibited a pattern of even greater downregulation of these signature cytokines than F1–04-A-G1, along with reduced expression of IFN-γ, IL-13, IL-18, IL-1α, and MCP-1 (*p* < .05 vs F1–04-A-G1) (Table 4). In summary, with the exception of 3A2, all anti-F1 mAbs shared the F1–04-A-G1 cytokine/chemokine signature and also showed significant bacterial aggregation (Figure 2d) and enhancement of phagocytosis (Figure 3a). Therefore, we predicted that 3A2 would provide little or no in vivo protection, but the other anti-F1 candidate antibodies might be effective at protecting animals from *Y. pestis* challenge.

**Table 3.** Fold change in cytokine levels 2 h post invasion with *Y. pestis pgm*- pPst- initially grown at 37°C preincubated with anti-F1 mAbs.

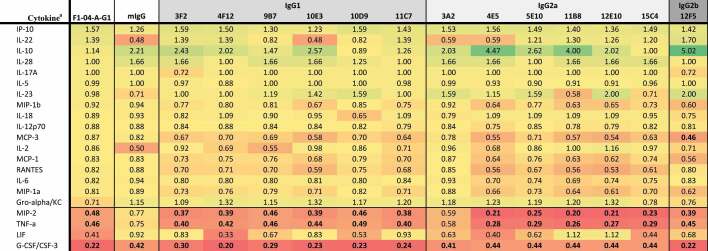

^a^The cytokine results are shown as the ratio of the levels expressed by Yp infected macrophages only and are based on the geometric mean (pg/mL). Cytokines where all values were identical, or all but one were identical, were omitted. Bolded values are up- or down-regulated compared to Yp only (up- or down-regulated >2-fold with *p*-value < .01).
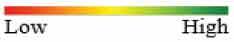

**Table 4.** Fold change in cytokine levels 24 h post invasion with *Y. pestis pgm*- pPst- initially grown at 37°C preincubated with anti-F1 mAbs.

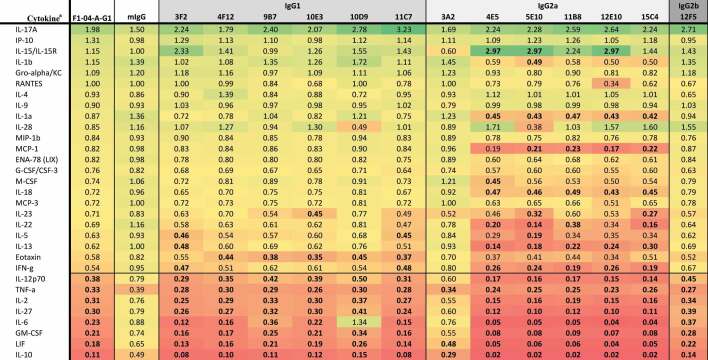

^a^The cytokine results are shown as the ratio of the levels expressed by Yp infected macrophages only and are based on the geometric mean (pg/mL). Cytokines where all values were identical, or all but one were identical, were omitted. Bolded values are up- or down-regulated compared to Yp only (up- or down-regulated >2-fold with *p*-value < .01).
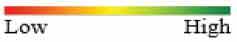

### In vitro results are predictive of in vivo efficacy in the mouse model of pneumonic plague

**Figure 4.**
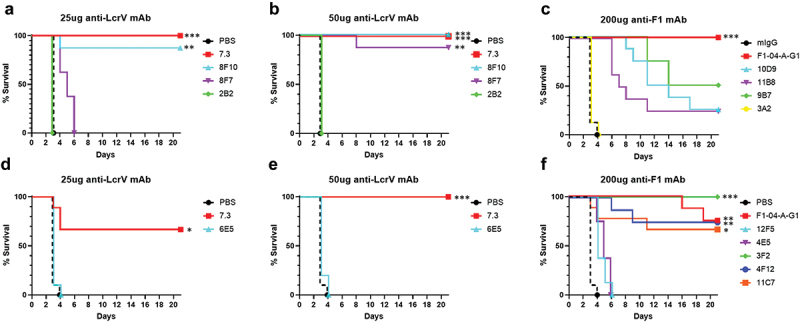
Survival curves of anti-LcrV and anti-F1 mAb treated BALB/c mice challenged with aerosolized *Y. pestis*. Mice were treated with 25 µg or 50 µg of anti-LcrV or 200 µg of anti-F1 mAbs per dose 18 h prior to whole body aerosol challenge with *Y. pestis* CO92. PBS or mouse polyclonal IgG (mIgG) was used as a control. a) Mice (*n* = 8/group) were exposed to ~19 LD_50_ (1.28x10^6^ cfu) of *Y. pestis* CO92 with the exception of the PBS control group with was exposed to ~13 LD_50_ (8.7x10^5^ cfu). b) Mice (*n* = 8/group) were exposed to ~13 LD_50_ (8.7x10^5^ cfu) of *Y. pestis* CO92. c) Mice (*n* = 8/group) were exposed to ~1 LD_50_ (8.17x10^4^ cfu) of *Y. pestis* CO92. d) Mice (*n* = 9 for 7.3; *n* = 10 for PBS and 6E5) were exposed to ~12 LD_50_ (7.95x10^5^ cfu) of *Y. pestis* CO92. e) Mice (*n* = 9 for 7.3; *n* = 10 for PBS and 6E5) were exposed to ~12 LD_50_ (7.95x10^5^ cfu) of *Y. pestis* CO92. f) Mice (*n* = 10 for PBS; *n* = 8 for the rest of the groups) were exposed to ~12 LD_50_ (7.95x10^5^ cfu) of *Y. pestis* CO92. *p*-values for survival rate comparisons groups are: *p < .01, **p < .001, ***p < .0002 (Comparisons show significant differences versus the PBS or mIgG control groups).

To determine which antibody-mediated characteristics are predictive of in vivo protection based on the in vitro assays described above, a diverse selection of mAbs were chosen to assess their protective efficacy in BALB/c mice relative to the two previously well-characterized protective mAbs, the anti-LcrV 7.3 and the anti-F1 F1–04-A-G1. Mice were administered, intraperitoneally, 25 µg/dose or 50 µg/dose of anti-LcrV, or 200 µg/dose of anti-F1 mAbs 18 h prior to exposure to aerosolized *Y. pestis* CO92. Either PBS (sham) or mouse polyclonal IgG (mIgG) was used as a control. Furthermore, in the interest of animal welfare we reduced the number of control animals (Figure 4a) and utilized the control group from a parallel experiment (Figure 4b) for statistical analysis; in addition, we referenced extensive historical data that demonstrates that sham treated animals succumb to infection at 3–4 days after exposure to aerosolized bacteria.^27,49^ Following the challenge, the mice were observed for 21 days.

Four novel anti-LcrV mAbs were compared to 7.3 to evaluate their protective efficacy after 21 days of challenge. Of the mice treated with anti-LcrV mAbs, similar levels of protection were observed with 25 µg/dose of 7.3 (100% and 67% survival, Figure 4a,b) and 8F10 (88% survival, Figure 4a) mAbs, reaching significance relative to the PBS control group as well as historical data. For 8F7 mAb, significant protection was only observed following 50 µg/dose treatment (88% survival), while the same dose conferred 100% protection with 7.3 and 8F10 (Figure 4b). Following 25 µg/dose treatment with 8F7 the mean time to death was significantly greater relative to the control groups (5 days vs 3.1 days, *p* < .0001), although all mice succumbed to disease or were euthanized in accordance with early endpoint euthanasia criteria (Figure 4a). No protection or enhancement in MTD was seen with two other anti-LcrV mAbs; 2B2 or 6E5 (Figure 4d,e).

Nine novel anti-F1 mAbs were compared to F1–04-A-G1 to evaluate protective efficacy following 21 days of challenge. Treatment with 200 µg/dose of F1–04-A-G1 resulted in 100% survival, while novel anti-F1 mAb 9B7 conferred 50% protection, and 11B8 or 10D8 only afforded 25% protection. Significance relative to PBS control was only reached following F1–04-A-G1 treatment. Administration of 3A2 was no different from control treated mIgG mice, with all mice succumbing to infection 4 days post-challenge (Figure 4c). In a separate challenge experiment, five additional anti-F1 mAbs were evaluated. Treatment with F1–04-A-G1 and 4F12 afforded 75% survival, although the MTD was greater following F1–04-A-G1 treatment (18.6 days vs 8.6 days), but the MTD difference did not reach statistical significance. A slightly lower level of protection was conferred by 11C7 mAb (67% survival), while 100% protection was afforded by 3F2 mAb (Figure 4f). Both 12F5 and 4E5 afforded no protection, but did significantly increase the MTD from 3.1 days to 4.5 and 5.1 days (*p* ≤ .0002 vs PBS), respectively.

## Discussion

The development of novel monoclonal antibodies directed against *Y. pestis* that can be used as prophylactics or therapeutics (alone or in combination with adjunct medical countermeasures) is critical for a rapid and effective medical countermeasure strategy. Production of novel mAbs directed against bacterial pathogens, such as *Y. pestis*, can be a lengthy process, and efficacy screening in animal models is required for most candidate antibodies due to the absence of reliable in vitro down-selection criteria. Here we describe a novel, in vitro, testing strategy for identifying both direct and indirect antibody-mediated responses in order to bridge in vitro functional data with in vivo protection data. This preliminary in vitro screening increases assay throughput and expedites the testing of antibodies, allowing for the efficient down-selection of the most promising candidates to be more rigorously tested in vivo.

Bacteria were grown under different temperature conditions in order to enrich for capsule production or T3SS expression and availability and incubated with anti-F1 or anti-LcrV mAbs.^10,50^ Appreciable bacterial aggregation was only observed with anti-F1 mAbs due to robust capsule formation, while, presumably, the relatively low number, of injectosomes (T3SS) on the surface of the bacteria resulted in no reproducible aggregation in the presence of anti-LcrV mAbs.^55,56^ Although some level of aggregate variability was observed within the anti-F1 antibody subclasses, the IgG1 antibodies appeared to induce higher aggregate formation relative to IgG2a antibodies (Figure 2c). In addition, all IgG1 antibodies tested in in vivo experiments provided some level of protection, while three out of four IgG2a or IgG2b antibodies did not, with the most opsonophagocytic IgG2a antibody (11B8) providing only 25% protection in vivo. Enhanced aggregate formation, along with IgG1 subclass designation, were important components of the overall antibody characterization and eventual down-selection of an anti-F1 mAb that enhanced in vivo protection.

Increased bacterial uptake by RAW264.7 cells was observed with anti-LcrV mAbs, which was associated with enhanced protection in vivo. However, IgG1 subclass antibody, 8F10, was more protective in mice even though the IgG2a antibody, 8F7, was more opsonophagocytic in vitro. Importantly, the commercially available normal mouse IgG shows significant opsonization when the bacteria are initially grown at 28°C but, as expected, offered no protection to the mice. Within our data set presented here, the IgG2a subclass mAbs appear to require greater level of opsonophagocytosis relative to IgG1 mAbs in order to partly compensate for subclass-specific limitations. Unlike anti-LcrV mAbs, there appeared to be no direct correlation between the level of enhanced opsonophagocytosis by anti-F1 mAbs and the extent of protection in mice. All anti-F1 mAbs significantly opsonized the bacteria indicating that a basal level of antibody-bacteria interaction is an important initial screen for antibody function and specificity, but not necessarily reflective of in vivo efficacy. Importantly, data collected for both F1 and LcrV targets demonstrate that opsonophagocytosis index, when used alone, does not appear to be reliably predictive of in vivo protection. Previous work has demonstrated that anti-LcrV antibodies promote phagocytosis of the bacteria, and the type and activation state of immune cells can result in differential impacts of the antibodies on in vitro experiments.^17,57,58^ Additionally, anti-LcrV antibodies can prevent the secretion of immune-regulatory Yops, directly result in altered host-cell immune responses, and may prevent early bacterial growth.^59–61^ These diverse and nuanced potential mechanisms of action of anti-LcrV antibodies further complicate the evaluation of mAb efficacy in vitro and thus, other parameters in addition to opsonophagocytic indices are required.

The protective cytokine profile following 2 h of macrophage infection in the presence of anti-LcrV mAbs was defined by the upregulation of G-CSF, LIF, MIP-1β, TNF-α, MIP-1α, MCP-3, and MIP-2α; this pattern was only observed with the 7.3, 8F10 and 8F7 mAbs. Twenty-four h after infection IL-10, LIF, M-CSF, IL-6, and IL-27 upregulation with the same mAbs was associated with protection in mice. Previous studies have shown that LcrV binding to the toll-like receptor 2 (TLR2) causes an immunosuppressive effect by enhancing IL-10 production.^18,62–64^ This is supported by the demonstration that IL-10 upregulation was seen only in the experiments with anti-LcrV antibodies and bacteria grown at 28°C (with a brief incubation at 37°C) for optimal T3SS injectisome availability. In experiments with anti-F1 antibodies, IL-10 was downregulated, potentially due to reduced TLR2-LcrV interaction and because the F1 capsule hinders TLR4 activation by LPS.^65^ The level of IL-10 upregulation coupled with increased levels of internalized bacteria at 2 h post-infection, as well as IgG1 subclass, appear to correlate with in vivo protection with anti-LcrV antibodies.

For the anti-F1 mAbs, protective efficacy appeared to be associated with downregulation of G-CSF, TNF-α and MIP-2 at 2 h post-infection. Furthermore, although all three cytokines were downregulated, the extent of downregulation was different between the IgG1 and IgG2a antibody subclasses. At 24 h post-invasion, the cytokine profile of the protective anti-F1 mAbs demonstrated the downregulation of IL-10, LIF, GM-CSF, IL-6, IL-27, IL-2, TNF-α, and IL-12p70. Again, there was a distinct cytokine signature and magnitude of cytokine inhibition associated with anti-F1 antibody subclasses; treatment with IgG2a mAbs was associated with more extensive downregulation of the aforementioned cytokines than IgG1 mAbs, or even the “benchmark” mAb F1–04-A-G1, but this increased inhibition was not associated with enhanced protection in mice.

Surprisingly, for both anti-F1 and anti-LcrV mAbs, the most protection was conferred to mice by IgG1 mAbs relative to IgG2a or IgG2b. It is widely accepted that mouse IgG1 antibodies are associated with a Th2-like immune response and have limited ability to fix complement, especially via the classical pathway.^66^ There is some evidence to suggest that mouse IgG1 molecules retain the ability to fix complement,^67^ but the dogma indicates that IgG2a is superior to IgG2b, which is superior to IgG1. With regard to intrinsic flexibility, murine IgG1 is assumed to be relatively rigid compared with other isotypes such as IgG2a in mice and IgG1 in humans.^68–70^ However, murine IgG1 antibodies overall may have greater antigen affinity relative to IgG2a.^71,72^ Higher affinity, based on the equilibrium dissociation constant, was also indicative of protection with anti-LcrV mAbs but not with anti-F1 mAbs.^45^ Recent reports also indicate that with increased somatic hypermutation comes increased antibody specificity but at the cost of impaired conformational stability. These observations were based on the lower melting temperatures which are an indicator of decreased thermodynamic stability and lower resistance to unfolding.^73^ The half-lives of IgG1 and IgG2a have been determined to be similar, between 6 and 8 days, while IgG2b was lower at 4–6 days.^74,75^

Interestingly, mouse IgG1 antibodies, relative to IgG2a/IgG2b, have poor antibody-dependent cellular cytotoxicity based on lower affinity for FcγR (FcγRI and FcγRIV) as well as complement-dependent cytotoxicity.^76–78^ In vivo, these properties of IgG2a might predispose *Y. pestis* to partially hijack antibody- and complement-mediated opsonization and thereby enhance host cell invasion and immune subversion. Thus, for IgG2a antibodies to confer protection they may need to have potent neutralizing activity that would mitigate the enhancement of cell invasion by Fc (such as FcγRIV) and complement receptor interactions. In addition, FcγRIV is present on neutrophils and has a much higher binding affinity to IgG2a relative to FcγRIIB and FcγRIII receptors, implying that IgG2a enhances neutrophil recruitment more than IgG1. Neutrophil recruitment is critical for bacterial clearance in the first 24 h following *Y. pestis* infection, but excessive inflammation would be detrimental to host survival.^79^

It is also plausible that the subclass-specific effects that we see differ between IgG1 and IgG2a/IgG2b may be directly modulated by bacterial factors in a way specific to *Y. pestis* infection, such as the observed effects of group A streptococci endoglycosidases.^80–82^ Bacterial Fc-binding proteins play a critical role in immune evasion, traditionally associated with gram-positive bacteria but also described in gram-negative bacteria, specifically in pathogenic *Y. pestis* species.^83,84^ Zav’yalov, et al. demonstrated that *Y. pestis* PsaA is able to bind to the human Fc regions of IgG subclasses with the exception of IgG4.^85^ Although no binding to mouse IgG was observed in that study, subversion of antibody function by binding to the Fc region is seen in many bacterial pathogens, and the role of *Y. pestis* PsaA (pH 6 antigen) is reminiscent of Protein A derived from *Staphylococcus aureus*.^86^ Interestingly, binding affinities of Protein A, as well as Protein G, are stronger for IgG2a and IgG2b than to IgG1.^87^ Thus, we hypothesize that if *Y. pestis* utilized similar immune evasion mechanisms it would be better able to subvert IgG2a/b activity and more susceptible to IgG1 antibodies.

The conformation and accessibility of the antigenic determinant plays a critical role in the induction of a proper antibody response. The mAbs, evaluated in this study, were produced from recombinant LcrV, F1 or the chimeric F1-V protein. In both the anti-F1 and the anti-LcrV mAb panels, the two least efficacious mAbs (3A2 and 2B2) were produced following F1-V immunization. While 2B2 possessed high affinity for LcrV, the 3A2 mAb directed against F1 was shown to have poor affinity for the recombinant F1 protein and did not bind to the F1 multimeric form.^45^ Although, F1-V vaccination is highly protective in various animal models, the fusion-protein clearly induces a fraction of the immune response that is directed against epitopes that are likely not found in the native proteome of *Y. pestis*.^25,27^ This further emphasizes the importance of current efforts toward optimizing the subunit vaccine formulations directed against *Y. pestis*.

In conclusion, we have further optimized a gentamicin protection assay to specifically examine novel anti-F1 and anti-LcrV monoclonal antibodies. By controlling the growth temperature and, thus, enriching for the expression of the known targets of novel mAbs, we were able to identify important in vitro characteristics that were associated with in vivo protection (Table 5). When taken together, our in vitro data generated with an attenuated strain of *Y. pestis* were able to predict in vivo protection in the mouse pneumonic plague model using the fully virulent *Y. pestis* CO92 strain. While this effort was focused on evaluating monoclonal antibodies against two very well-characterized virulence factors that are known to generate a protective immune response, we believe this assay and the downstream analyses will be useful when examining novel antibodies directed against other bacterial antigens that are less understood or protective antigens that have yet to be identified.

**Table 5.** Summary of in vitro and in vivo data for each antibody characterized in this study. The anti-LcrV antibodies are listed, followed by the anti-F1 antibodies. For in vivo survival, the positive controls (7.3 and F1–04-A-G1) were measured twice, with the second experiment in parentheses. Mean time-to-death or euthanasia is expressed as the mean of all animals in the group, measured in days post-challenge. “na” indicates that the mean time-to-death or euthanasia is not applicable because all animals survived to the end of the study. Font color denotes ≥ 50% (green) or ≤ 50% (red) survival at the highest dose tested.

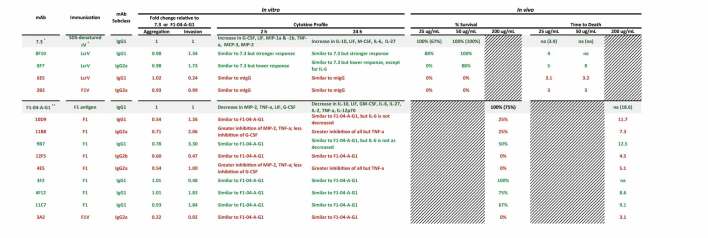

*Hill, Jim, et al. “Regions of *Yersinia pestis* V antigen that contribute to protection against plague identified by passive and active immunization.” Infection and immunity 65.11 (1997): 4476-4482. **Anderson Jr, George W., et al. “Protection of mice from fatal bubonic and pneumonic plague by passive immunization with monoclonal antibodies against the F1 protein of *Yersinia pestis*.” The American journal of tropical medicine and hygiene 56.4

## Supplementary Material

Supplemental Material

## Data Availability

Data will be made available without unnecessary delay.
