# Supplementary material for: Functional assays to screen and select monoclonal antibodies that target Yersinia pestis
Source: Hum Vaccin Immunother. 2023 Jun 8;19(2):2216085. doi: 10.1080/21645515.2023.2216085 (PMC10332183; doi:10.1080/21645515.2023.2216085)
Supplement: Supplemental Material [file KHVI_A_2216085_SM9597.pdf]

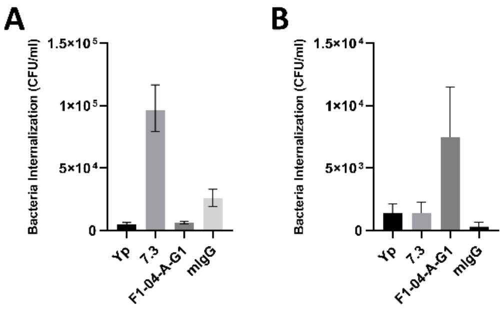

<u>Figure S1.</u> Optimization of macrophage infection conditions for *Y. pestis*. *Y. pestis pgm*- pPst- initially grown at **(A)** 28°C or **(B)** 37°C on SBA for 24 h followed by a switch to a liquid BHI medium and grown for an additional 2 h at 37°C. RAW264.7 cells were infected, at an MOI of 10, with *Y. pestis* (Yp) alone or with *Y. pestis* pretreated for 1 h with mAbs (F1-04-A-G1, 7.3) or the mouse non-specific polyclonal IgG (mIgG) control at 10 μg/mL. The number of viable bacteria (Geometric Mean and Geometric Standard Error are depicted) recovered after 2 h of infection from one representative experiment (n = 6) are shown.
